# Anti-glomerular basement membrane disease—treatment standard

**DOI:** 10.1093/ndt/gfaf190

**Published:** 2025-09-18

**Authors:** Stephen P McAdoo, Charles D Pusey

**Affiliations:** Vasculitis Centre, Dept of Immunology & Inflammation, Imperial College London, London, UK; Vasculitis Clinic, Department of Renal Medicine, Imperial College Healthcare NHS Trust, London, UK; Vasculitis Centre, Dept of Immunology & Inflammation, Imperial College London, London, UK; Vasculitis Clinic, Department of Renal Medicine, Imperial College Healthcare NHS Trust, London, UK

**Keywords:** ANCA, crescentic glomerulonephritis, immunosuppression, plasma exchange, vasculitis

## Abstract

We review the current standards of treatment and discuss novel developments in the pathophysiology, diagnosis, outcome prediction and management of anti-glomerular basement membrane (anti-GBM) disease. Anti-GBM disease is a unique form of small vessel vasculitis affecting the glomerular and pulmonary capillaries. It is caused by autoantibodies directed against the α3 chain of type IV collagen, leading to rapidly progressive glomerulonephritis with pulmonary haemorrhage in ∼50% of cases. Diagnosis relies on clinical features, kidney biopsy showing linear IgG deposition along the GBM, and/or detection of circulating anti-GBM antibodies. Historically, untreated disease was rapidly fatal, but the introduction of plasma exchange combined with cyclophosphamide and glucocorticoids has significantly improved outcomes, particularly in patients who are not dialysis-dependent at presentation. Dialysis-dependent patients have a lower likelihood of renal recovery, and treatment decisions must consider biopsy findings, clinical severity and potential contraindications to standard immunosuppression. Unlike anti-neutrophil cytoplasm antibody (ANCA)-associated vasculitis, relapses are rare in classic anti-GBM disease, and long-term maintenance immunosuppression is not routinely required. However, ‘double positive’ patients (anti-GBM and ANCA) have a higher relapse risk and require maintenance immunosuppressive treatment. Atypical anti-GBM presentations, including seronegative cases, are now better recognized but their optimal management remains unclear. Future research should define the use of oral versus intravenous cyclophosphamide in anti-GBM disease, clarify the role of rituximab and determine the place of emerging therapies such as imlifidase. Advances in risk stratification and ongoing trials are expected to inform treatment individualization and to improve treatment approaches for this aggressive autoimmune disease.

Box 1: **‘In a Nutshell’**

• Further support for plasma exchange (PEX) and immunoadsorption: recent large cohort studies reinforce PEX as an essential treatment in anti-glomerular basement membrane (anti-GBM) disease together with cyclophosphamide and glucocorticoids. Immunoadsorption is emerging as an alternative, offering comparable antibody clearance, when available.

• Rituximab expands treatment options: mounting experience with rituximab suggests favourable outcomes in selected cases and provides an alternative for those with contraindications to cyclophosphamide or in refractory disease.

• Atypical and variant anti-GBM disease: increasing recognition of atypical presentations (e.g. IgA-dominant disease, seronegative anti-GBM) requires clinicians to maintain a high index of suspicion and adapt diagnostic and therapeutic strategies accordingly.

• Imlifidase offers a new therapeutic approach: imlifidase shows promise for rapid clearance of circulating and tissue-bound anti-GBM antibodies, with encouraging Phase 2 outcomes.

## INTRODUCTION

Anti-glomerular basement membrane (anti-GBM) disease is a unique form of immune-complex small vessel vasculitis characterized by the presence of circulating and tissue-bound IgG autoantibodies directed against basement membrane antigens, particularly the non-collagenous domain of the α3 chain of type IV collagen [α3(IV)NC1] [1, 2]. These antibodies mediate severe glomerular and alveolar injury, leading to rapidly progressive glomerulonephritis (RPGN) and, in 40%–60% of cases, pulmonary haemorrhage.

Current standard of care includes plasma exchange (PEX) to remove pathogenic autoantibodies, along with cyclophosphamide and glucocorticoids to suppress ongoing antibody production and to attenuate tissue inflammation [3]. This therapeutic approach, first introduced in the 1970s, is supported by observational studies and clinical experience, rather than prospective randomized controlled trials (RCT). Emerging strategies—including immunoadsorption, rituximab and imlifidase—have been evaluated in selected cohorts, but their role remains adjunctive or investigational at present.

Treatment decisions in anti-GBM disease must consider the severity of renal and pulmonary involvement, histological findings, serological features such as anti-neutrophil cytoplasm antibody (ANCA) co-positivity, and potential contraindications to standard immunosuppression. In addition, specific clinical scenarios, including *de novo* or recurrent disease post-transplant, and treatment of anti-GBM during pregnancy or in childhood, require individualized management.

This review summarizes current evidence and practical recommendations for the management of anti-GBM disease, with emphasis on therapeutic principles, treatment indications and futility, and evolving approaches in specific patient populations.

## TREATMENT STANDARD

Rapid and accurate diagnosis is essential to guide appropriate therapy in anti-GBM disease, and where there is a high index of clinical suspicion and evidence of immediately organ- or life-threatening disease, empirical treatment (e.g. with glucocorticoids and/or plasmapheresis) may be initiated whilst waiting for definitive diagnostic tests. A comprehensive review of diagnostic approaches, however, is beyond the scope of this review [4, 5]; important considerations are summarized in Table 1. The term ‘atypical anti-GBM disease’ has recently emerged to describe cases with classic linear immunoglobulin G (IgG) staining on renal biopsy, but no detectable circulating antibody or otherwise uncharacteristic clinical, immunological or pathological features. This rare phenomenon has been reviewed recently [6], and optimal treatment approaches are not well defined. Therefore, this treatment standard will focus on the management of ‘classic’ anti-GBM disease.

**Table 1:** Diagnostic considerations in anti-GBM disease.

	‘Classic’ anti-GBM disease	‘Atypical’ anti-GBM disease
Description	• Circulating and tissue-bound anti-GBM IgG and presentation with RPGN (>90%) and alveolar haemorrhage (40%–60%)	• Recently emerged to describe cases with classic linear IgG staining on kidney biopsy, but without identifiable circulating Ab and other uncharacteristic clinical or pathological features
Serology	• Circulating Ab to α3(IV)NC1 in >90%	• Circulating Ab to α3(IV)NC1 absent by standard assays
	• Commercial immunoassays have high sensitivity and specificity	• Currently unclear whether this reflects true seronegativity, different GBM antigens, or lack of appropriate detection methods
	• Patients may demonstrate circulating Ab to other type IV collagen chains (e.g. α5 chain in ∼70%) but these are not tested in routine clinical practice	
	• 30%–40% double-positive for circulating ANCA	
Immunohistology	• Detection of deposited polyclonal IgG by IF on frozen kidney tissue is diagnostic ‘gold standard’, typically in strong linear ribbon-like pattern	• Linear IgG along GBM in the absence of another known cause (e.g. diabetes, fibrillary GN, monoclonal immunoglobulin deposition diseases)
	• Immunoperoxidase techniques may be less sensitive	• Deposited Ab show light chain restriction in ∼50%
	• Immunohistology may also show deposited complement components (e.g. C3)	• No deposits on EM
	• No deposits on EM, unless concomitant glomerular pathology (e.g. membranous GN, IgA nephropathy)	
Glomerulonephritis	• Diffuse crescentic GN in >80%	• Mesangial or endocapillary proliferative GN most common
	• Average proportion of affected glomeruli is ∼75%	• Focal crescents and fibrinoid necrosis ∼10%
	• Crescents typically of uniform age; mix of acute and chronic lesions (or extraglomerular vasculitis) suggests concomitant ANCA-GN	• Secondary focal-segmental glomerulosclerosis common
Alveolar haemorrhage	• No formal diagnostic criteria; should be differentiated from infections, pulmonary oedema and uraemic haemoptysis in setting of oligoanuric kidney injury	• Typically absent
	• Chest CT typically shows diffuse bilateral ground glass opacifications or frank consolidation, often with apical and peripheral sparing	
	• Bronchoalveolar lavage typically shows increasing blood content on serial washes; haemosiderin-laden cells on cytological examination	
	• Increased KCO may have positive predictive value, but may not be achievable in critically unwell patients	
Relapse and recurrence	• Relapse and allograft recurrence are rare (provided transplantation performed in the absence of circulating Ab)	• Possible, chronic course more common
		• Recurrent disease after kidney transplantation is recognized

Ab, antibody; CT, computed tomography, EM, electron microscopy; GN, glomerulonephritis; IF, immunofluorescence; KCO, diffusion capacity for carbon monoxide.

**Figure 1:**
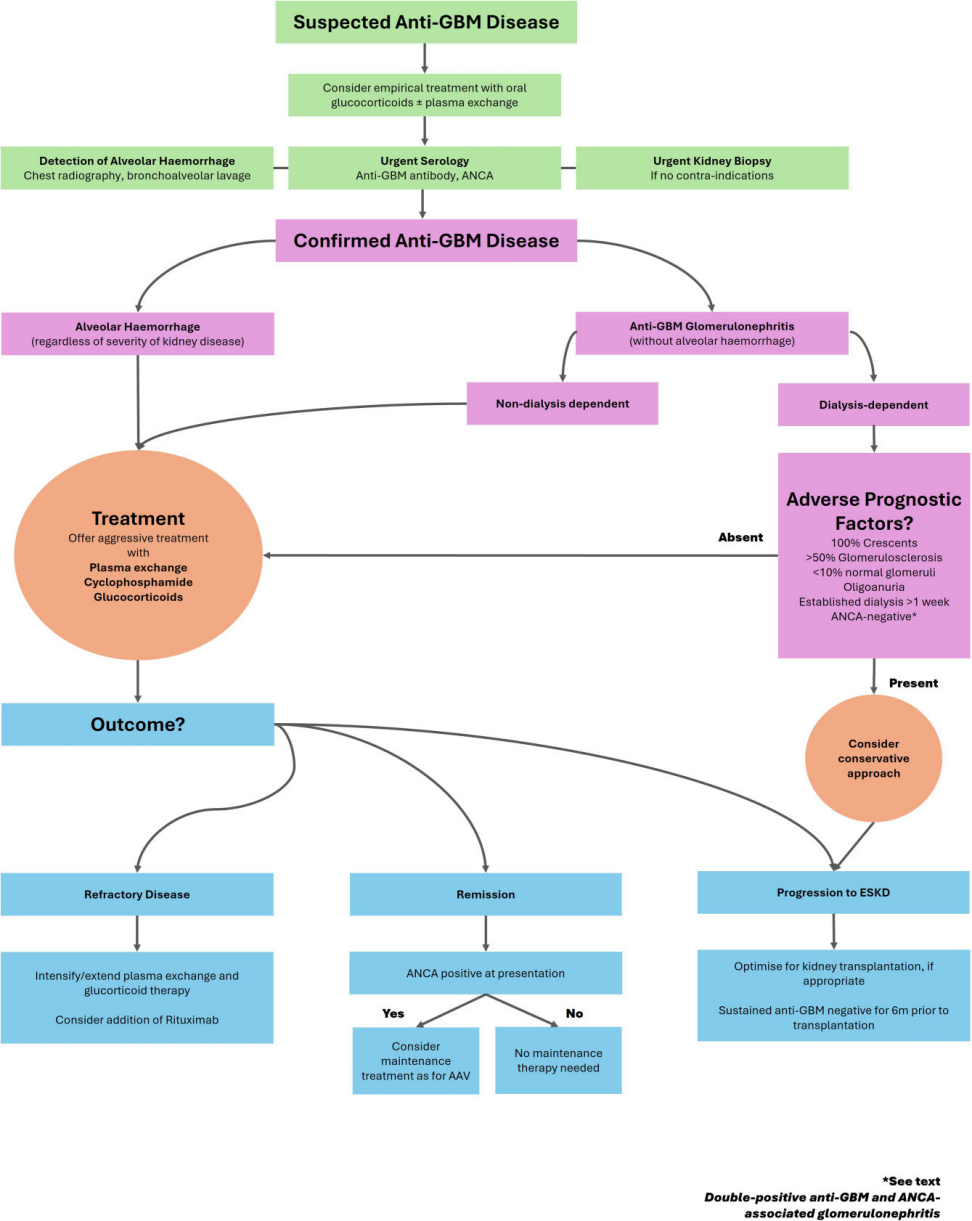
Treatment algorithm for anti-GBM disease.

A treatment algorithm is presented in Fig. 1, and a detailed description of therapeutic agents is provided in Table 2.

**Table 2:** Conventional and novel therapeutic agents in anti-GBM disease.

Agent, mechanism of action	Dose, duration and markers of response	Toxicities and cautions
PEX		
• Typically removes 50%–60% of intravascular IgG per session (for a 1.0–1.5 plasma volume exchange) • ∼45% of total body IgG is intravascular at baseline; after PEX, IgG re-distributes from the extravascular compartment into plasma over 6–24 h, thus daily treatment is recommended	• Daily 40–60 mL/kg exchange for 5% human albumin solution • Add fresh human plasma (300–600 mL) within 3 days of invasive procedure (e.g. kidney biopsy) or in patients with alveolar haemorrhage • Continue until antibody levels are fully suppressed, typically 14 days • Monitor antibody levels regularly during and after cessation of treatment; PEX may require reinstatement if antibody levels rebound	Monitor and correct as required: • Platelet count; aim >70 × 10^9^/L • Fibrinogen; aim >1 g/L; may require cryoprecipitate supplementation • Haemoglobin, aim for >90 g/L • Corrected calcium; aim to keep in normal range Access-related complications • Infection • Venous thromboses
Cyclophosphamide		
• A prodrug that requires metabolism by hepatic cytochrome P450 enzymes to active metabolites (e.g. 4-hydroxycyclophosphamide, aldophosphamide, phosphoramide mustard; these act as alkylating agents that inhibit leucocyte proliferation	• 2–3 mg/kg/day (ideal body weight; maximum 200 mg) orally for 2–3 months • Consider reduced dose in patients >55 years or with dialysis-dependent kidney injury • Administer dose after haemodialysis or PEX treatment • Limited evidence for the use of pulsed IV therapy	• Bone marrow suppression—stop if leucocyte count falls <4 × 10^9^/L and restart at reduced dose when recovered • Urothelial toxicity—encourage adequate hydration and frequent voiding in non-oliguric patients; chelation therapy generally not required • Infertility—consider gamete preservation in younger patients if feasible • Secondary malignancy (long-term)
Rituximab		
• Chimeric IgG1 mAb against CD20; leads to depletion of circulating B lymphocytes	• Limited evidence for first-line use, but may be considered where there are contraindications to cyclophosphamide or as adjunct in severe or refractory disease • 2× 1 g at day 0 and 14 OR 375 mg/m^2^ weekly ×4 • PEX should be delayed for 24–48 h after rituximab infusion; adequacy of peripheral B-cell depletion may be determined by CD19 count	• Infusion reactions • Hypogammaglobulinaemia—intravenous Ig supplementation (administered after PEX treatment) may be considered
Glucocorticoids		
• Broad immunosuppressive and anti-inflammatory effects	• Prednisolone 1 mg/kg/day (maximum 60 mg) orally • Reduce dose weekly to 20 mg by 6 weeks, then gradually taper until complete discontinuation at 6 months • Limited evidence to support the use of pulsed intravenous methylprednisolone, provided other components of therapy are initiated rapidly	• Infection—increased risk of bacterial, fungal and opportunistic infections • Endocrine and metabolic—hyperglycaemia, new-onset or worsening diabetes, weight gain, adrenal suppression • Cardiovascular—hypertension, dyslipidaemia, fluid retention • Musculoskeletal—osteoporosis, fracture risk, avascular necrosis, myopathy • Gastrointestinal—gastritis, peptic ulcer disease • Neuropsychiatric—insomnia, altered mood, psychosis • Ophthalmologic—cataracts, glaucoma
Imlifidase		
• Cleavage of all circulating (and potentially tissue bound) IgG at the hinge region into F(ab′)2 and Fc fragments • Rapid onset of action (2–6 h)	• Currently under investigation in a global, Phase 3 RCT • 0.25–0.5 mg/kg single dose • PEX should not be administered for >48 h after administration (MW ∼61 kDa, present unbound in plasma and may undergo non-specific removal) • PEX may be required if Ab levels rebound after treatment	• Infusion reactions • Profound hypogammaglobulinaemia; total circulating IgG begins to recover >1 week; additional prophylactic antibiotic therapy may be indicated • Avoid co-administration of human (or rabbit) mAb for >4 days (mean elimination half-life of imlifidase is ∼96 h)
Prophylactic treatments	• For oropharyngeal fungal infection (e.g. nystatin, amphotericin, or fluconazole) while on high-dose glucocorticoids • For peptic ulcer disease (e.g. with proton-pump inhibitor) while on high-dose glucocorticoid treatment • For *Pneumocystis jiroveci* pneumonia (e.g. cotrimoxazole) while receiving high-dose glucocorticoids and cyclophosphamide • For osteoporosis (e.g. calcium and vitamin D supplementation) for those receiving high-dose glucocorticoids; bisphosphonates likely to be contraindicated due to impaired eGFR • Consider prophylaxis against HBV reactivation (e.g. lamivudine) in patients who have evidence of previous infection (e.g. HBV cAb positive)	

Ab, antibody; cAb, core antibody; HBV, hepatitis B virus; IV, inrtavenous; mAb, monoclonal antibody; MW, molecular weight.

### Plasma exchange

Given the direct pathogenicity of anti-GBM antibodies, their rapid removal with PEX is a central component of therapy. In Lockwood’s seminal 1976 study, the use of PEX was associated with rapid removal of circulating autoantibodies, resolution of lung haemorrhage, and salvage of kidney function in patients who were not requiring dialysis at presentation [7]. A subsequent small RCT likewise showed a more rapid fall in anti-GBM titres when PEX was added to immunosuppressive therapy, with a trend towards improved renal outcome (although groups were poorly matched based on histopathological severity) [8]. The early adoption of PEX was further supported by several observational studies that suggested improved renal and patient survival compared with historic cohorts treated with immunosuppression alone [9].

More recent observational studies support the continued use of PEX in anti-GBM disease. A French multicentre study of 122 patients with severe disease (median creatinine 7 mg/dL; 78% pulmonary–renal syndrome; 68% dialysis-dependent) treated with PEX and immunosuppression reported 1-year survival of 87% [10]. In multivariable analysis, a higher number of exchanges improved survival [hazard ratio (HR) 0.87 per session; 95% confidence interval (CI) 0.77–0.98], with eight sessions predicting favourable outcomes (positive and negative predictive value 95% and 47%, respectively). A second French study of 119 patients (median age 54 years, median creatinine 7.2 mg/dL, 78% requiring dialysis) similarly showed excellent survival at 1 and 5 years (95% and 92%, respectively) [11]. PEX (used in >80%) was associated with improved survival (HR 0.29; 95% CI 0.08–0.98). In a large Chinese cohort (*n* = 448, mean creatinine 9.2 mg/dL), 56% received PEX alongside glucocorticoids and cytotoxics [12]. Mortality fell dramatically over three decades (12-month mortality from 57.1% to 6.9%), paralleling greater use of PEX and earlier diagnosis. PEX was independently associated with reduced mortality (HR 0.30; 95% CI 0.16–0.95). Another retrospective study of 107 patients with anti-GBM disease treated in West China likewise found that treatment with PEX was independently associated with improved in-hospital outcomes [mortality and dialysis-dependency at discharge (HR 0.18; 95% CI 0.05–0.63)] and long-term survival [at 2 years (HR 0.15; 95% CI 0.04–0.55)] [13]. Notably, patients who initiated PEX early had better prognoses. The study also suggested that 5–10 PEX sessions might suffice for maximal risk reduction. Finally, a Japanese nationwide database study (*n* = 207) of patients with anti-GBM disease presenting with dialysis-dependent kidney failure, but without diffuse alveolar haemorrhage, found that the addition of PEX to glucocorticoid therapy was associated with a significant reduction in in-hospital mortality (10.7% versus 28.2%) [14]. This study underscores the potential benefit of PEX for survival, even in patients with severe renal disease.

Although these retrospective studies have clear limitations, including variable use of concurrent immunosuppressants, lack of predictive histopathological data, and risk of confounding by indication, they consistently indicate that inclusion of PEX in the early treatment of anti-GBM is associated with improved outcomes. Both the American Society for Apheresis (Grade 1C–2B) and the Kidney Disease: Improving Global Outcomes (KDIGO) guidelines recommend inclusion of PEX in therapeutic regimens for anti-GBM disease [3, 15]. Either centrifugal or plasma filtration methods can be used, as IgG is effectively cleared by both techniques. A recommended treatment schedule and practical considerations are summarized in Table 2.

### Immunoadsorption

Immunoadsorption is an alternative form of pheresis that may be more efficient than PEX for the removal of pathogenic IgG, although it may not remove other pro-inflammatory or pro-coagulant factors that contribute to the renal injury. A recent small study compared the kinetics of autoantibody removal by immunoadsorption and PEX in 38 patients with anti-GBM disease and/or ANCA-associated vasculitis (AAV) [16], finding that modalities were comparable in reducing pathogenic autoantibody levels over seven sessions. Immunoadsorption achieved a greater reduction in total IgG while preserving other immunoglobulins (IgA, IgM), and without significant differences in clinical outcomes. A nationwide study from Germany compared clinical outcomes of >900 patients with anti-GBM disease treated with either immunoadsorption or PEX [17]. Noting that a minority of patients in the study (∼11%) were treated with immunoadsorption, this was associated with comparable overall and kidney survival compared with those treated with PEX, but with reduced risk of extrapulmonary bleeding and requiring blood transfusion. These findings support the consideration of immunoadsorption as an alternative to PEX in anti-GBM disease, particularly in settings where immunoadsorption is readily available and when preservation of plasma components is desired.

### Cyclophosphamide

Cyclophosphamide is the recommended first-line immunosuppressive treatment for anti-GBM disease. As with PEX, this recommendation is based on retrospective and observational studies that show improved patient survival and renal outcomes when cyclophosphamide was used (in combination with PEX and glucocorticoids) compared with treatment with glucocorticoids alone [18].

Daily oral cyclophosphamide (e.g. 2–3 mg/kg/day; maximum 200 mg) is the most frequently used approach in anti-GBM disease [9]. In older adults and dialysis-dependent patients, many centres initiate reduced oral doses (e.g. 50–100 mg/day), adjusted for tolerability and renal function. In AAV, the equivalence of daily oral and pulsed intravenous cyclophosphamide in induction therapy was established in a large RCT [19]. However, this study excluded patients with severe disease requiring dialysis or mechanical ventilation and those receiving PEX, thus findings cannot be confidently extrapolated to the more severe setting of anti-GBM disease, in which disease mechanisms differ. The parent drug cyclophosphamide has low molecular weight (∼261 Da) and is water soluble, meaning that it is theoretically dialysable or removable by PEX before metabolism (to its active metabolites, which are more polar, bind to tissues and are less easily removed).

Thus, daily oral dosing may provide more consistent drug exposure than with pulsed intravenous dosing in patients who are receiving extracorporeal therapies. There are, however, few data comparing the use of oral versus intravenous cyclophosphamide in anti-GBM disease. One retrospective cohort analysis suggests that daily oral cyclophosphamide may be associated with improved survival compared with pulsed intravenous treatment [10].

Since nearly all published experience in anti-GBM disease has used daily oral dosing, and for the reasons outlined above, we recommend this as the first-line approach in this disease, and this is consistent with current KDIGO recommendations. In addition, because the risk of relapse of anti-GBM is very low, and ∼3 months of cytotoxic therapy is usually required, concerns about total cumulative dose of cyclophosphamide are perhaps less relevant than in AAV. Prescribing considerations are summarized in Table 2.

### Rituximab

Rituximab is an effective treatment for several autoantibody-associated glomerular diseases though published experience in anti-GBM disease is limited. A recent international review identified 67 patients (including 14 paediatric cases) who received rituximab either as first-line (*n* = 39) or second-line (*n* = 28) therapy [20]. Most patients also received PEX (93%) and glucocorticoids (98%), and 54% received cyclophosphamide at some point. Overall, patient survival was high at 91%, and kidney survival was 67%, with better outcomes in patients treated with rituximab as second-line therapy compared with first-line (73% versus 46%, *P* = .03). Rituximab appeared to have a favourable safety profile, with 16% of patients experiencing transient adverse events, mostly of minor severity. However, evidence for use of rituximab in anti-GBM disease remains relatively limited and at risk of publication bias. While rituximab may rapidly deplete circulating CD20+ B cells, it will not affect other cell types that contribute to disease (e.g. T cells, neutrophils, monocytes/macrophages), unlike cyclophosphamide. Thus, at present, there is insufficient evidence to recommend its use first-line in the treatment of anti-GBM disease. It may have a role, however, where there are compelling contraindications to cyclophosphamide therapy, or as adjunctive treatment in severe disease or to reduce cumulative cyclophosphamide exposure, or to lower antibody levels pending kidney transplantation. Since rituximab is likely to be removed by PEX, administration should be carefully timed and PEX delayed for 24–48 h after rituximab infusion if possible. Administration of additional rituximab infusions after completion of PEX treatment may also be considered.

### Alternative immunosuppression

The use of other immunosuppressive therapies in anti-GBM disease is not well described. Mycophenolate mofetil (MMF) and cyclosporine treatment is reported in individual cases or small series [21–23], but there is insufficient evidence to recommend their use in first-line therapy. These agents may, however, have a role in extended immunosuppressive treatment in rare cases of persistent anti-GBM positivity after completion of cyclophosphamide therapy.

### Glucocorticoids

Glucocorticoid therapy is typically with oral prednisolone, initiated at 1 mg/kg/day (maximum 60 mg daily) and reduced weekly to 20 mg by 6 weeks, then gradually tapered to completed discontinuation by 6 months. This regimen may be adjusted depending on clinical response and/or occurrence of refractory disease. In our experience, high-dose intravenous glucocorticoids are not required, provided the other components of therapy, including PEX and cyclophosphamide, can be initiated promptly [9]. Reduced-dose glucocorticoid regimens, which have gained traction in AAV, have not been tested in anti-GBM disease and cannot be assumed to be effective. Likewise, there is no evidence for the use of novel glucocorticoid-sparing therapies such as avacopan, although this may be considered, in addition to standard glucocorticoid treatment, in patients with double-positive ANCA and anti-GBM disease.

## TREATMENT INDICATIONS, FUTILITY AND OUTCOMES

Diffuse alveolar haemorrhage in anti-GBM disease may be life-threatening but is highly treatment responsive, with 90%–100% achieving remission with immunosuppression and plasmapheresis [9, 24]. Therefore, treatment is always recommended when alveolar haemorrhage is present, regardless of the presence or severity of kidney disease.

In patients with RPGN, dialysis status at treatment initiation is the most significant determinant of long-term kidney outcomes. Long-term follow-up of a large cohort (*n* = 71) treated consistently with PEX, cyclophosphamide and glucocorticoids found that independent kidney function was maintained in most patients who did not require dialysis at presentation: renal survival at 1 and 5 years was 95% and 94%, respectively, with presenting creatinine <500 µmol/L, and 82% and 50%, respectively, for creatinine >500 µmol/L [9]. In dialysis-dependent patients, however, only 8% recovered independent kidney function at 1 year. Similarly low rates of recovery from dialysis-dependent kidney injury are consistently reported in more recent series [10, 11, 25, 26], and optimistic rates of recovery approximate ∼17%–20% at best [27, 28].

The duration of dialysis requirement at the time treatment initiation may also be important. In our experience, patients requiring dialysis for >7 days before initiation of immunosuppressive therapy very rarely recover independent kidney function, whereas those with a dialysis duration of <72 h may still do so, particularly in the absence of adverse histological features (see below). We therefore recommend that the timing of dialysis initiation and its underlying indication (e.g. hyperkalaemia versus anuria) be carefully considered when evaluating the potential for renal recovery and when deciding whether to proceed with full immunosuppressive therapy.

Several studies have sought to identify additional clinical or histopathological predictors of recovery. In the Levy series, no dialysis-dependent patient with 100% glomerular crescents recovered kidney function [9]. A larger multicentre biopsy study (*n* = 123) confirmed that dialysis-dependence, fewer normal glomeruli and greater interstitial infiltrate predict end-stage kidney disease (ESKD). No recovery was observed with 100% crescents or >50% glomerulosclerosis [26]. A small UK study further identified oligoanuria as the strongest predictor of dialysis non-recovery [25].

We therefore recommend intensive treatment for anti-GBM glomerulonephritis when there is:

- Clinically evident alveolar haemorrhage (regardless of renal function);
- Rapidly progressive glomerulonephritis (RPGN) without immediate dialysis requirement;
- RPGN requiring dialysis, if:
  - <100% glomerular crescents
  - <50% glomerulosclerosis
  - Non-oliguric presentation
  - Alternative biopsy findings to account for dialysis requirement (e.g. severe acute tubular injury),
  - Recent dialysis initiation (e.g. <72 h)
  - Coexisting ‘double-positive’ ANCA disease (see later)

Rare cases of recovery despite adverse features, however, support individualized treatment decisions [29, 30]. A short trial of therapy may be reasonable, particularly in younger patients who may tolerate treatment with lower risk of adverse events, and this can be tapered if no renal recovery is evident within 2–4 weeks. Short-term immunosuppression may also aid antibody clearance to facilitate earlier kidney transplantation.

## SUPPORTIVE CARE

In patients presenting with severe disease, immediate organ support may be required. Approximately 50% patients will have an indication for acute renal replacement therapy at diagnosis. One small series suggests that 11% of patients presenting with alveolar haemorrhage require artificial ventilation [24]. In severe lung haemorrhage, extracorporeal membrane oxygenation may be considered, and appears to be associated with favourable outcome despite the requirement for systemic anticoagulation in the setting of alveolar bleeding [4]. Alveolar haemorrhage may be exacerbated by pulmonary irritants including respiratory infection and pulmonary oedema, and these should be aggressively managed. Patients receiving cytotoxic and high-dose glucocorticoids treatment should receive routine prophylactic treatments to mitigate significant risk of adverse events, including infection and metabolic disorders (Table 2).

## RELAPSE AND LONG-TERM OUTCOMES

Relapse is rare in anti-GBM disease, occurring in <3% of patients [9]. It is usually associated with ongoing exposure to pulmonary irritants such as cigarette smoke or hydrocarbons [31, 32], and avoidance of these precipitants is an essential part of long-term management. We recommend repeat kidney biopsy in cases of relapse with kidney involvement, to secure an accurate diagnosis and to exclude concomitant pathologies such as AAV and membranous nephropathy. In confirmed cases, standard re-treatment with PEX, cytotoxics and glucocorticoids is usually indicated.

The long-term outcome of patients who progress to ESKD due to anti-GBM glomerulonephritis is comparable to ESKD of other causes, whether they remain on dialysis or undergo transplantation [33, 34]. It is important, however, that transplantation is not performed in the presence of circulating anti-GBM antibodies, as this is associated with a high likelihood of disease recurrence, at frequencies of up to 50% in historical series [35]. A period of at least 6 months of sustained seronegativity is advised prior to undertaking transplantation in patients who have reached ESKD due to anti-GBM disease. In these circumstances, with current immunosuppressive regimens, recurrent disease is rare [33, 36, 37].

## SPECIAL CIRCUMSTANCES

### Refractory anti-GBM disease

Refractory anti-GBM disease is a term used to describe the persistence of active disease despite standard therapy, usually associated with ongoing production of pathogenic autoantibodies. It is uncommon but carries a high risk of progression to ESKD and/or mortality due to persistent pulmonary haemorrhage. There is, however, no accepted definition of ‘refractory’ anti-GBM disease, and it must be distinguished from irreversible kidney damage (most commonly due to delayed diagnosis) and/or pulmonary haemorrhage mimics (e.g. infection, pulmonary oedema, uraemic haemoptysis, progression to acute respiratory distress syndrome). Given the increased risk of venous thromboembolism during active systemic vasculitis, pulmonary embolism should be considered [38], as should development of alternative primary lung pathology, such as pulmonary fibrosis, particularly in ANCA-positive patients. Where refractory anti-GBM disease is suspected, management strategies include intensification of PEX (e.g. increasing session frequency or) dose or glucocorticoid treatment, and consideration of adjunctive therapies such as rituximab, which can more effectively deplete CD20+ B cells responsible for ongoing antibody production. In patients with refractory disease and irreversible renal injury, transitioning to supportive care and planning for renal replacement therapy may be appropriate. Given the potential for persistent anti-GBM antibody production to delay eligibility for transplantation, ongoing immunosuppression aimed at antibody clearance may still be beneficial even in the absence of functional kidney recovery.

### Double-positive anti-GBM and ANCA-associated glomerulonephritis

Circulating ANCA are commonly found in patients with anti-GBM disease, occurring in 30%–40% of cases, usually recognizing myeloperoxidase. It has been shown that ANCA may be detected before the onset of anti-GBM disease [39], suggesting that ANCA-induced glomerular inflammation may be a trigger for the development of an anti-GBM response, perhaps by modifying or exposing usually sequestered disease-associated epitopes in GBM. A large European study found that double-positive cases have the age distribution of AAV patients, with prodromal symptoms and more chronic lesions on renal biopsy, supporting the hypothesis that AAV is the initial pathology [40]. At diagnosis, however, double-positive cases had the severe features of anti-GBM disease, with high rates of alveolar haemorrhage and dialysis-dependent renal failure, and so these cases should receive standard initial treatment for this condition. A subgroup of double-positive patients may, however, have a greater propensity to recovery from dialysis (33% versus 17% in double- and single-positive cases, respectively, in this study) which is more in keeping with AAV. These cases should be considered for intensive treatment, even when dialysis-dependent, and particularly if kidney biopsy findings indicate alternative mechanisms of kidney injury (e.g. pauci-immune glomerulonephritis with circulating anti-GBM antibodies). In addition, during long-term follow up (median 4.8 years), patients with double-positive disease relapsed at a frequency comparable to those with AAV, whereas no single-positive anti-GBM cases relapsed. Thus, double-positive patients should receive maintenance immunosuppression as for AAV, especially when prodromal or extra-renal features are present at diagnosis.

### Anti-GBM disease associated with membranous nephropathy

There are several reports of anti-GBM disease associated with membranous nephropathy, occurring as a preceding, simultaneous or succeeding diagnosis [41, 42]. As for the association with ANCA, it is postulated that disruption of glomerular architecture by one disease reveals hidden epitopes that allow the second process to occur. A rapid decline in kidney function in a patient with known membranous nephropathy should raise suspicion of the development of superimposed anti-GBM disease or another crescentic nephritis, and re-biopsy is recommended. We suggest that such cases are treated initially as for anti-GBM disease, since the outcome of patients treated before the onset of dialysis-dependent renal failure appears to be favourable as in typical anti-GBM disease [43]. Of note, anti-GBM antibodies are not a common finding in patients with systemic lupus erythematosus, with or without membranous glomerulonephritis [44].

### IgA-dominant anti-GBM disease

IgA-mediated anti-GBM disease is a rare isotypic variant of classic anti-GBM disease in which the pathogenic autoantibodies are predominantly of the IgA type, rather than IgG. Descriptions are limited to case reports [45]. This entity is characterized histologically by strong linear deposition of IgA along the GBM on immunofluorescence microscopy, with minimal or absent IgG staining. Patients typically present with RPGN, like classic anti-GBM disease, although pulmonary involvement appears to be less frequent. Circulating anti-GBM antibodies may not be detectable by conventional assays optimized for IgG (but may be identified using dedicated approaches) and diagnosis requires renal biopsy with appropriate immunostaining for IgA. Due to its rarity, evidence to guide treatment is limited, but most reported cases have been managed with PEX, glucocorticoids and cytotoxic agents, following protocols for IgG-mediated disease, although renal outcomes may be poorer [45], perhaps reflecting the lack of an easily measured circulating biomarker to guide treatment and monitoring. Awareness of this variant is important, particularly with regards to planning kidney transplantation in apparently seronegative patients.

### Isolated pulmonary involvement in anti-GBM disease

Presentation with isolated or predominant pulmonary involvement in anti-GBM disease is uncommon, occurring in <10% of patients in larger series [46, 47]. Some patients may have mild urinary abnormalities and minor proliferative changes on renal biopsy, but with preserved excretory function, while others may have no clinical or histological evidence of renal inflammation. Renal biopsy, however, may still reveal linear deposits of immunoglobulin, including in those patients who are negative by serological assay, and may therefore have diagnostic value. A small series from Sweden described four young female patients who presented with severe alveolar haemorrhage and non-severe renal disease, who were seronegative for circulating anti-GBM antibodies by conventional assay [48]. They were, however, found to have circulating IgG4 anti-GBM antibodies by dedicated enzyme-linked immunosorbent assay, and this was confirmed on kidney biopsy. Together, these findings suggest that clinical presentation in anti-GBM disease may be influenced by differences in antibody subclass or antigen target and highlight the need to consider variant anti-GBM disease in cases of ‘idiopathic’ pulmonary haemorrhage. We suggest conventional intensive treatment with PEX and immunosuppression in such cases.

### Atypical anti-GBM disease

Important diagnostic considerations related to ‘atypical’ anti-GBM disease are summarized in Table 1 [6, 49, 50]. It is not clear whether these cases represent distinct clinical entities, or less advanced cases on the spectrum of disease severity, and strict classification criteria are lacking; indeed IgA- and IgG4-mediated disease and isolated lung disease have been described as ‘atypical’ in some series. Around 50% of patients with atypical anti-GBM disease have restriction for either kappa or lambda light chains on immunofluorescence testing, and the recent description of recurrent monotypic atypical anti-GBM disease in kidney allografts suggests that this phenomenon is due to a circulating monoclonal protein in at least some cases [51]. However, the typical ultrastructural deposits seen in monoclonal immunoglobulin deposition disease or proliferative GN with monoclonal immunoglobulin deposits are not observed and—in the reported series—these patients did not have an identified plasma cell dyscrasia [49]. Of note, however, a separate report described a case of florid anti-GBM disease with kappa-light chain restriction on GBM staining in association with a circulating paraprotein [52], thus suggesting that anti-GBM disease may occur within the spectrum of ‘monoclonal gammopathy of renal significance’ and that evaluation for an underlying plasma cell disorder should be considered. Treatment of atypical anti-GBM disease is not well defined, as patients generally have milder renal involvement and better outcomes compared with classic disease. Immunosuppression (e.g. with glucocorticoids, cyclophosphamide or MMF) may be considered based on the severity of glomerular injury, but plasma exchange is generally not indicated in the absence of RPGN or an identified circulating autoantibody [49, 50].

### Post-transplant anti-GBM disease in Alport syndrome

Kidney transplant recipients with Alport syndrome may mount an alloimmune response to neo-antigens contained in normal α3, α4 or α5 chains found in the allograft. In classical X-linked Alport syndrome (caused by mutations in the *COL4A5* gene encoding the α5 collagen chain) these antibodies do not recognize the same epitopes of the α3 chain as in typical anti-GBM disease patients, but rather a distinct, composite epitope on the α5 chain [53]. Commercially available anti-GBM assays, which are optimized to detect reactivity to the α3(IV)NC1 antigen, may fail to detect circulating antibodies in this setting. Anti-GBM antibodies may be detected in 5%–10% of Alport patients following transplantation, although the development of overt glomerulonephritis is less frequent (perhaps owing to the effects of maintenance immunosuppression). When glomerulonephritis develops, however, it usually occurs early and carries a high risk of allograft loss [54, 55]. Repeated transplantation in this setting almost invariably leads to more aggressive disease recurrence and rapid graft loss and is undertaken at very high risk [56]. Individuals with large *COL4A5* gene deletions are at increased risk of post-transplant anti-GBM disease, and recent guidelines encourage the use of genetic testing to inform discussions regarding the risk of *de novo* anti-GBM disease after transplantation [57].

### Anti-GBM disease in pregnancy

Anti-GBM disease related to pregnancy is rare; a systematic review in 2014 identified eight cases in the literature [58], and there are a small number of subsequent case reports [59–61]. Disease most commonly occurs in the second trimester, and while six of eight cases in the review resulted in live births, the rate of adverse foetal and maternal outcomes was high. Glucocorticoids and plasma exchange may be employed safely in pregnancy, and judicious use of cyclophosphamide may be considered from the second trimester, when the risk of foetal abnormalities is reduced after organogenesis. Azathioprine may be used as an alternative immunosuppressant, though it is unlikely to be sufficiently potent for early disease control, and rituximab may also be considered. Transplacental transfer of ANCA has been implicated in neonatal renal–pulmonary syndrome, and it is notable that anti-GBM antibodies were demonstrated in two of five neonates tested in the anti-GBM disease series, though none had evidence of kidney or lung disease, perhaps reflecting the increased expression of the α1α1α2 collagen IV protomer (rather than α3α4α5) *in utero* and in neonatal GBM.

### Anti-GBM disease in children

Anti-GBM disease is rare in children, accounting for <5% of paediatric crescentic glomerulonephritis [62]. Clinical presentation is often severe, with a high incidence of dialysis-dependence at diagnosis. Specific treatment data in children are lacking, and most paediatric series have used adult treatment protocols [63], although careful adjustment of PEX volumes and cytotoxic drug dosing is required. Outcomes in children appear to mirror those in adults: renal recovery is possible if therapy is initiated before the onset of dialysis-dependence, but prognosis is poor in cases presenting with established kidney failure and extensive crescent formation. Long-term complications of cyclophosphamide, including infertility and secondary malignancy, are a particular concern in paediatric patients. Rituximab with MMF has been tested as an alternative to cyclophosphamide in a small number of cases, without dialysis-dependence, and outcomes appeared favourable [64]. Management in experienced centres and careful long-term follow-up, including monitoring for growth or fertility issues, are essential components of care.

A summary of strategies to personalize the treatment of anti-GBM disease is presented in Box 2.

Box 2: **Strategies to personalize treatment.**

- Histological severity: patients with fewer crescents, limited glomerulosclerosis, and more preserved glomeruli benefit most from aggressive treatment; biopsy findings, together with clinical features, should guide treatment decisions in dialysis-dependent cases.
- ANCA positivity: double-positive patients tend to be older and have increased relapse risk. They require acute anti-GBM therapy initially, followed by maintenance immunosuppression similar to ANCA-associated vasculitis treatment.
- Atypical or seronegative presentations: if circulating anti-GBM antibodies are negative, but kidney biopsy shows linear IgG, treatment is still likely indicated; immunosuppression alone (without plasma exchange) may be sufficient in mild or slowly progressive cases.
- Contraindications to standard therapy: rituximab may be considered for patients with severe cytopenias, malignancy or fertility concerns precluding cyclophosphamide.
- Transplantation planning: anti-GBM antibodies should be undetectable for at least 6 months prior to kidney transplantation to prevent recurrence in the allograft.

## NEW DEVELOPMENTS

### Pathophysiology

In the glomerular and alveolar basement membrane the predominant chains of type IV collagen are α3, α4 and α5. These combine to form a triple helical protomer which links end-to-end via their non-collagenous (NC1) domains to form a hexamer. The major target antigen in anti-GBM disease [α3(IV)NC1] is normally sequestered in the NC1 hexamer, which is stabilized by sulfilimine crosslinking. Two major B-cell epitopes at the amino terminus of the α3 chain have been identified, E_A_ (17–31) and E_B_ (127–141), and levels of antibodies to these epitopes correlate with disease severity.

Studies using anti GBM antibodies eluted from the kidneys of patients with anti-GBM disease have shown that while all bind to the α3 chain, some also bind to the α4 and α5 chains [53]. It has been suggested that this is due to intermolecular epitope spreading, as has been demonstrated in rodent models of the disease. Autoantibodies to α5 are more common than those to α4, and epitopes E_A_ and E_B_ on α5, homologous to those on the α3 chain, have been identified. The α5 E_A_ epitope is recognized by antibodies in some patients with X-linked Alport syndrome after transplantation. Interestingly, a distinct genetic variant of the α3 chain (the Zurich variant), which alters the function of the NCI hexamer, has been found both in familial anti-GBM disease and in autosomal Alport syndrome [65].

The formation of stabilizing sulfilimine bonds in the NC1 hexamer is dependent on the enzyme peroxidasin. Recent studies have shown the presence of inhibitory anti-peroxidasin antibodies in ∼50% of anti-GBM patients, and it was suggested that these may lead to exposure of the usually cryptic autoantigen on the α3 chain to the immune system [66].

The GBM also contains several laminin isoforms, in particular laminin 521. Autoantibodies to laminin 521 were demonstrated in >30% of a cohort of anti-GBM patients and were associated with a higher incidence of alveolar haemorrhage, suggesting that they may be involved in causing lung injury [67].

As expected in an autoimmune disease, there are strong associations between anti-GBM disease and major histocompatibility complex (MHC) genes, in particular HLA DRB1*1501 (DR15), and there are several other positive and protective associations. Using DR15–α3_(135–145)_ tetramers, it was shown that patients with anti-GBM disease had 100-fold more specific CD4 T cells than DR15-positive controls. Whether antigen presentation on different MHC molecules may determine the nature of immune response to α3 was then investigated [68]. In a transgenic mouse model, when an α3 epitope was presented by HLA DR15 then proinflammatory T cells were generated and infiltrated the kidney causing glomerulonephritis, whereas when presented by HLA DR1 then regulatory T cells were produced and no glomerulonephritis occurred.

### Outcome prediction

A recent retrospective, multicentre study assessed predictors of kidney outcomes in 174 patients with anti-GBM disease (to date, the largest histopathological study in this disease) [69]. The study validated the Renal Risk Score—originally developed for AAV—and demonstrated it could effectively stratify renal prognosis in anti-GBM disease. Overall, the two strongest independent predictors of ESKD were the need for dialysis at presentation and the percentage of normal glomeruli on kidney biopsy, with a 10% cutoff providing meaningful risk separation. Patients with >10% normal glomeruli and no need for dialysis had excellent 3-year renal survival (∼96%), while those needing dialysis and with <10% normal glomeruli had very poor outcomes (∼14%). The findings support using simple, early clinical and biopsy features to guide management decisions and highlight the importance of biopsy even in severe disease. This risk stratification tool could help individualize therapy and improve kidney outcomes in anti-GBM patients.

### Treatment

IdeS (IgG-degrading enzyme of *Streptococcus pyogenes*) is an endopeptidase that enables microbial evasion of the host immune response by rapidly cleaving all IgG subclasses at the hinge region into F(ab′)2 and Fc fragments. Recent studies have aimed to exploit this unique activity for the treatment of antibody-mediated diseases, including HLA-incompatible renal transplantation. In a murine model of anti-GBM disease, IdeS treatment reduced glomerular inflammatory cell infiltration and albuminuria [70]. Treatment was notable for cleaving GBM-bound IgG—thus affecting non-circulating antibodies that may possibly be inaccessible to plasma exchange. In a preliminary clinical study of three patients, a single dose of IdeS was remarkably efficient at clearing circulating and tissue-bound anti-GBM antibodies, though rebound of circulating IgG occurred after approximately 1 week in all patients, and none recovered kidney function [71].

A subsequent Phase 2 study investigated imlifidase in 15 patients with severe anti-GBM disease and estimated glomerular filtration rate (eGFR) <15 mL/min/1.73 m^2^. A single dose of imlifidase led to rapid clearance of circulating anti-GBM antibodies within 6 h [72]. Patients also received standard treatment with cyclophosphamide and glucocorticoids, and PEX was added in cases of antibody rebound; this occurred in 10 patients at a median of 6.5 days. At 6 months, 67% of patients were alive and dialysis-independent, a significantly better outcome compared with 18% in matched historic controls. Treatment was generally safe, with no major adverse events attributed to imlifidase. A global, open-label, Phase 3 RCT (the GOOD-IDES-02 study, NCT05679401) completed recruitment of 50 patients this year, and will compare imlifidase plus standard of care, versus standard of care alone, in patients with entry eGFR <20 mL/min/1.73 m^2^. The primary objective is to assess the impact of imlifidase on kidney function, assessed by eGFR and dialysis independence, at 6 months. Results are anticipated in late 2025 and, if positive, may support regulatory approval and broader clinical use; however, the cost of imlifidase may limit its widespread adoption.

## SUMMARY

Anti-GBM disease remains a uniquely aggressive autoimmune condition, and rapid diagnosis and decisive therapy are critical to preserving life and organ function. Over recent decades, the standard of care—plasma exchange combined with immunosuppressive therapy—has improved outcomes significantly. Despite progress, several areas require further study. There is little evidence for the benefit of high-dose intravenous glucocorticoids, although they are often used despite their adverse effects. The optimal route of cyclophosphamide administration (oral versus intravenous) remains unclear in anti-GBM disease, particularly in patients receiving extracorporeal therapies. More robust data are needed on the efficacy and safety of rituximab, particularly its role as a first-line agent. The ongoing GOOD-IDES-02 Phase 3 trial will clarify whether imlifidase improves outcomes compared with standard therapy alone, particularly in patients presenting with adverse prognostic factors for renal recovery. Indeed, more prospective data are required to refine treatment futility criteria and to guide individualized therapy in dialysis-dependent patients. In addition, better understanding of the clinical significance and management of atypical anti-GBM disease presentations, including IgA-dominant and seronegative disease, is needed. Future advances in immunopathology, biomarker development, and targeted therapies offer hope for improving prognosis and reducing treatment-related toxicity in this rare autoimmune disease.

## Data Availability

This article is a review and does not contain any original research data. All data discussed are publicly available in the cited literature.
